# Draft Genome Sequence of Extended-Spectrum-β-Lactamase-Producing *Escherichia coli* Strain CCUG 62462, Isolated from a Urine Sample

**DOI:** 10.1128/genomeA.01382-16

**Published:** 2016-12-15

**Authors:** Anna Johnning, Hedvig E. Jakobsson, Fredrik Boulund, Francisco Salvà-Serra, Edward R. B. Moore, Christina Åhrén, Nahid Karami, Erik Kristiansson

**Affiliations:** a Department of Mathematical Sciences, Chalmers University of Technology, Gothenburg, Sweden; b Centre for Antibiotic Resistance Research (CARe) at University of Gothenburg, Gothenburg, Sweden; c Department of Infectious Diseases, Sahlgrenska Academy, University of Gothenburg, Gothenburg, Sweden; d Department of Biology, University of the Balearic Islands, Palma de Mallorca, Spain; e Swedish Strategic Programme Against Antibiotic Resistance, Västra Götaland, Sweden

## Abstract

The draft genome sequence has been determined for an extended-spectrum-β-lactamase (ESBL)-producing (*bla*_CTX-M-15_) *Escherichia coli* strain (CCUG 62462), composed of 119 contigs and a total size of 5.27 Mb. This *E. coli* is serotype O25b and sequence type 131, a pandemic clonal group, causing worldwide antimicrobial-resistant infections.

## GENOME ANNOUNCEMENT

The *Escherichia coli* sequence type 131 (ST131) has emerged as a globally dispersed pathogen, the predominant lineage causing extraintestinal infections, and is often resistant to antibiotic treatment (1). The *E. coli* ST131 clonal group carries a multitude of resistance and virulence genes, including extended-spectrum β-lactamases (ESBLs). The ESBL-producing *E. coli* strain E10394 (= CCUG 62462) was isolated from a urinary tract infection (UTI) sample as part of an outbreak of ESBL-producing *Enterobacteriaceae* in a neonatal postsurgery ward at Sahlgrenska University Hospital, Gothenburg, Sweden, in 2008 (2). The strain was characterized as resistant to cefotaxime, tobramycin, and trimethoprim but susceptible to ciprofloxacin, and it was defined as an ESBL-producing strain. It was further characterized as sequence type 131 and serotype O25b. The strain was cultured on blood agar medium, and genomic DNA was isolated from bacterial biomass using a PureLink genomic DNA minikit (Invitrogen, Carlsbad, CA, USA). The genome was sequenced with an Illumina MiSeq instrument (Genomics Core Facility at University of Gothenburg), generating 1,427,585 paired-end reads of 250 bp, yielding a total of 357 Mb. The sequence reads were trimmed using Trim Galore! version 0.3.7 (http://www.bioinformatics.babraham.ac.uk/projects/trim_galore/) with command line arguments “—stringency 3—retain_unpaired.” After quality filtering, 1,424,760 paired reads and 2,356/75 unpaired forward/reverse reads were retained for analysis. The high-quality reads were assembled *de novo* with SPAdes version 3.7.0 (3), with command line arguments “—careful—cov_cutoff 5.” The resulting assembly consisted of 119 contigs, with a total assembly length of 5.27 Mb. The longest contig measured 515,034 bp, the *N*_50_ was 328,690 bp, and the average genome coverage of the assembly was 64×. The G+C content of the genome sequence was estimated at 50.8%. The genome sequence was annotated using the NCBI Prokaryotic Genome Annotation Pipeline (PGAP) (4); 5,104 coding sequences and 80 tRNAs were identified. Using ResFinder (5), a number of antibiotic resistance genes were found: *bla*_CTX-M-15_ on contig LZET01000074, *bla*_TEM-1_ on contig LZET01000061, *aac(3)-IIa* on contig LZET01000076, and *aadA5*, *mph*(A), *sul1*, and *dfrA17* on contig LZET01000047. PlasmidFinder (6) identified the following plasmid marker genes: *IncFIA* (nucleotide accession no. AP001918) on contig LZET01000040, *IncFIB* (accession no. AP001918) on contig LZET01000042, and *IncFII* (accession no. AY458016) on contigs LZET01000050, LZET01000068, and LZET01000072. In addition, genes involved in type IV secretion systems (T4SS) were identified, using CONJscan (7), included genes belonging to the mating-pair formation family F (MPF_F_), relaxases (MOB_F_, MOB_P_, and MOB_Q_), and type IV coupling proteins (T4CP). Three small plasmids were completely assembled. First, a 5,627-bp plasmid (contig LZET01000060), which was 99% identical to the pJJ1886_3 plasmid previously described in *E. coli* strain JJ1886 (accession no. CP006787.1), was found. Second, a 5,166-bp plasmid (contig LZET01000063), and last, a 1,718-bp plasmid (contig LZET01000087), which was 99% identical to plasmid pEC648 previously described in *E. coli* strain ST648 (accession no. CP008716.1), were found.

### Accession number(s).

This whole-genome shotgun project has been deposited at DDBJ/ENA/GenBank under the accession no. LZET00000000. The version described in this paper is version LZET00000000.1.
